# Impact of Low Maternal Weight on Pregnancy and Neonatal Outcomes

**DOI:** 10.1210/jendso/bvae206

**Published:** 2024-11-25

**Authors:** Nikhita Chahal, Tanya Qureshi, Soukaina Eljamri, Janet M Catov, Pouneh K Fazeli

**Affiliations:** Department of Medicine, Division of Endocrinology and Metabolism, Neuroendocrinology Unit, University of Pittsburgh School of Medicine, Pittsburgh, PA 15213, USA; Department of Medicine, Division of Endocrinology and Metabolism, Neuroendocrinology Unit, University of Pittsburgh School of Medicine, Pittsburgh, PA 15213, USA; Department of Medicine, Division of Endocrinology and Metabolism, Neuroendocrinology Unit, University of Pittsburgh School of Medicine, Pittsburgh, PA 15213, USA; Department of Obstetrics, Gynecology & Reproductive Sciences and the Department of Epidemiology, Magee-Womens Research Institute, University of Pittsburgh, Pittsburgh, PA 15213, USA; Department of Medicine, Division of Endocrinology and Metabolism, Neuroendocrinology Unit, University of Pittsburgh School of Medicine, Pittsburgh, PA 15213, USA

**Keywords:** underweight, body mass index, pregnancy, birth weight, gestational age

## Abstract

**Objective:**

To examine the effect of underweight maternal body mass index (BMI) on pregnancy complications and neonatal outcomes.

**Design:**

Cohort study.

**Setting:**

Tertiary academic center.

**Patients:**

A total of 16 361 mothers who delivered a singleton between 2015-2021 with either a BMI <18.5 kg/m^2^ (n = 732) or normal BMI (18.5 ≥ BMI <23 or 25 kg/m^2^, n = 15 629) at the initial prenatal visit or within 6 months of the initial visit.

**Main Outcome Measures:**

Birthweight, gestational age, neonatal intensive care unit admission, preterm birth, and fetal death; obstetrical complications including preeclampsia/eclampsia, premature rupture of membranes, preterm premature rupture of membranes, and postpartum hemorrhage.

**Results:**

Underweight women were younger and less likely to have private insurance (*P* < .01 for both) than normal-weight women. Approximately 23% of infants born to underweight mothers were small for gestational age and 15% were low birth weight vs 13.5% and 9% of infants of normal-weight mothers, respectively (*P* < .01 for both). These differences remained significant after adjusting for potential confounders. In adjusted logistic regression models, underweight women had a decreased risk of premature rupture of membranes and postpartum hemorrhage compared to normal-weight women.

**Conclusion:**

Underweight BMI during pregnancy is associated with an increased risk of small for gestational age and low birth weight infants and a decreased risk of premature rupture of membranes and postpartum hemorrhage. These findings suggest underweight BMI during pregnancy increases the risk of adverse neonatal outcomes, while maternal-related pregnancy outcomes are less affected.

Approximately 4% to 8% of women of reproductive age are classified as being underweight, defined as a body mass index (BMI) <18.5 kg/m^2^ [1, 2]. A subset of women also meet the *Diagnostic and Statistical Manual of Mental Disorders*, fifth edition criteria [3] for the diagnosis of anorexia nervosa. Anorexia nervosa is an eating disorder characterized by caloric restriction or excess calorie expenditure due to a distorted self-perception surrounding body weight, typically resulting in an inappropriately low body weight [3]. Low body weight is not without long-term implications that include low bone mineral density and impaired hematopoiesis due to hormonal adaptations from undernutrition [4-6]. It is estimated that about 1% to 3% of women suffer from an eating disorder in their lifetime with a 7.5% estimated prevalence in pregnant women [4, 7-10]. Furthermore, maternal anorexia nervosa has a prevalence rate of 0.05% to 0.50% in specific European and Caucasian populations [11].

While numerous studies have investigated the association between maternal overweight/obesity and adverse neonatal outcomes and pregnancy complications [12, 13], the effects of eating disorders and maternal underweight on such outcomes are less understood. Maternal underweight BMI has been associated with an increased risk of preterm birth, small for gestational age (SGA), as well as low birth weight [1, 12], but a major limitation of prior studies is the definition of underweight BMI that often includes women of normal BMI (BMI: ≥18.5 kg/m^2^) [14-16]. Additionally, retrospective and prospective studies report conflicting evidence of no differences or decreased risk of adverse neonatal and pregnancy outcomes in maternal low-weight [17-25] and eating disorder [26-30] mother-singleton pairs, and findings are potentially inconsistent between studies due to differences in comparison groups with very few studies using a normal-BMI referent group.

Therefore, low maternal BMI is an important modifiable and understudied risk factor [31, 32] and remains a public health concern as no prior studies have investigated the association between low-weight women and pregnancy/neonatal outcomes in a carefully defined population using strict BMI categories. In this study, we sought to understand the impact of maternal low weight on neonatal outcomes and pregnancy complications when compared with those in normal-weight mother-infant pairs using objective BMI assessments and controlling for maternal smoking, concomitant depression, and chronic illness—factors known to affect pregnancy/neonatal outcomes but not well accounted for in previous studies.

## Methods

Our study population consisted of women with singleton gestations delivered at Magee-Womens Hospital (University of Pittsburgh, Pittsburgh, PA, USA) from January 2015 to May 2021. Using the Steve N. Caritis Magee Obstetric Maternal and Infant (MOMI) database, a total of 16 361 women were identified and met our inclusion criteria of either low BMI (BMI <18.5 kg/m^2^) or normal BMI (18.5> BMI <23 or 25 kg/m^2^); Asian women with BMI ≥ 23 kg/m^2^ were excluded. Twin and high-order births were also excluded. If participants had multiple deliveries within the period of interest, only the first delivery was used in our analysis. This study was reviewed by the University of Pittsburgh Institutional Review Board.

Women were categorized as either low weight (BMI <18.5 kg/m^2^) or normal weight (18.5 ≥ BMI <23 or 25 kg/m^2^) according to World Health Organization BMI standards. For Asian women, normal BMI was defined as 18.5 ≥ BMI <23 kg/m^2^. Maternal BMI was obtained from the MOMI database, and for all low-weight women (n = 732), the medical record was independently reviewed, and BMIs were verified with anthropometric data in the electronic medical record to ensure BMIs were <18.5 kg/m^2^ at the time of the initial prenatal visit or within 6 months, either before or after, from the initial prenatal visit.

Adverse neonatal outcomes and pregnancy/delivery complications were compared in low- vs normal-weight women and their offspring. All outcome data were obtained from the MOMI database. Neonatal outcomes included birth weight, preterm birth, size for gestational age, neonatal intensive care units (NICU) stay, and fetal death. Low birth weight was defined as <2500 grams and preterm birth was defined as gestational age <37 weeks. Size for gestational age based on the Alexander National Fetal Growth Curve [33] was categorized as large for gestational age (LGA) and SGA if size for gestational age was >90th or <10th percentile, respectively. NICU stay and fetal death were defined as any admission ≥1 day or death of a liveborn neonate <7 days old, respectively.

Pregnancy and delivery complications included gestational diabetes mellitus, preeclampsia/eclampsia, premature rupture of membranes (PROM), preterm premature rupture of membranes (PPROM), and postpartum hemorrhage. PROM is defined as rupture of membranes prior to labor, which included women with rupture of membranes at less than, equal to, or greater than 37 weeks. PPROM is defined as rupture of membranes at less than 37 weeks only. Hypertensive disorders in pregnancy included women who were diagnosed with mild or severe preeclampsia, hemolysis, elevated liver enzymes, low platelet count syndrome, eclampsia, and chronic hypertension with preeclampsia. Women without hypertensive disorders or with transient or unspecified hypertension during pregnancy were included in the referent group.

Maternal characteristics (age, race, marital status, health insurance, smoking during pregnancy, maternal depression, parity, and prepregnancy diagnoses of diabetes mellitus and hypertension) and infant characteristics (birth weight, gestational age, and sex) were obtained from the MOMI database.

### Statistical Analyses

Wilcoxon rank sum, chi-square, and Fisher's exact tests were used to determine differences in baseline characteristics by maternal BMI. Logistic regression models were used to estimate adjusted odds ratios (aOR) and 95% confidence intervals (CI) for dichotomous outcomes (ie, low birth weight, preterm birth, LGA, SGA, NICU stay, fetal death, gestational diabetes mellitus, preeclampsia, PROM, PPROM, postpartum hemorrhage) associated with maternal BMI. Using covariates selected a priori, we adjusted for maternal age, race, marital status, health insurance, smoking during pregnancy, maternal depression, parity, and prepregnancy diagnoses of diabetes mellitus and hypertension. All testing was performed at a 2-sided *P*-value <.05 level of statistical significance using JMP Pro version 15.2.0 (SAS Institute, Cary, NC, USA).

## Results

### Characteristics of the Study Population

Approximately 4.5% of women had a low BMI in our study population (Table 1). Median BMI in underweight women was 17.8 (interquartile range: 17.2, 18.2) kg/m^2^ compared with 22.1 (interquartile range: 20.7, 23.5) kg/m^2^ in normal-weight women (data not shown). Low-weight women were younger and less likely to be married or have private insurance compared with normal-weight women. Maternal depression during pregnancy did not differ between low-weight and normal-weight women; however, low-weight women were more likely to be smokers and nulliparous compared with the normal-weight group. Approximately 22% of the low-weight mothers were African American and 17.5% were Asian compared with 13.8% and 7.3% of normal-weight mothers. There was no difference in prepregnancy diagnoses of diabetes mellitus and hypertension among women in the low-weight and normal-weight groups. Infants of low-weight women had lower birth weights and gestational ages at delivery compared with infants born to normal-weight women (Table 1).

**Table 1.** Baseline characteristics by maternal body mass index

Characteristics	No. of observations (n = 16 361)	Low-weight BMI (n = 732)	Normal-weight BMI*^a^* (n = 15 629)	*P*-value
Mothers				
Age (years)*^b^*	16 361	27 (22-31)	30 (26-33)	**<**.**01**
Race				**<**.**01**
Caucasian	10 665	372 (59.6)	10 293 (78.8)	
African American	1963	139 (22.3)	1824 (13.8)	
Asian	1073	109 (17.5)	964 (7.3)	
Pacific Islanders	66	1 (0.2)	65 (0.5)	
Indigenous persons	59	3 (0.5)	56 (0.4)	
Married	8560	311 (44.6)	8249 (54.7)	**<**.**01**
Privately insured	10 898	389 (53.1)	10 509 (67.2)	**<**.**01**
Smoking during pregnancy	1640	95 (13.5)	1545 (10.2)	**<**.**01**
Maternal depression during pregnancy	2314	104 (14.2)	2210 (14.1)	.96
Nulliparous	10 847	524 (71.6)	10 323(66.1)	**<**.**01**
Obstetrical complications				
Gestational diabetes mellitus	611	24 (3.3)	587 (3.8)	.51
Hypertensive disorders in pregnancy				.30
Gestational hypertension	1079	38 (5.2)	1041 (6.7)	
Mild preeclampsia	450	16 (2.2)	434 (2.8)	
Severe preeclampsia/HELLP*^c^*	551	27 (3.7)	524 (3.4)	
PROM	1971	71 (9.7)	1900 (12.2)	.05
PPROM	667	31 (4.2)	636 (4.1)	.83
Postpartum hemorrhage	603	22 (3.0)	581 (3.7)	.32
Prepregnancy conditions				
Diabetes mellitus	116	3 (0.4)	113 (0.7)	.49*^d^*
Hypertension	315	14 (1.9)	301 (1.9)	.98
Infants				
Males	8354	367 (50.5)	7987 (51.8)	.49
Birthweight (grams)*^b^*	16 144	3035 (2740-3340)	3250 (2920-3560)	**<**.**01**
Gestational age (weeks)*^b^*	16 358	39.3 (38.1-40.1)	39.4 (38.4-40.3)	**.01**
NICU admission	2400	116 (16.0)	2284 (14.8)	.39
Fetal death	119	5 (0.7)	114 (0.7)	1.0*^d^*

Abbreviations: BMI, body mass index; HELLP, hemolysis, elevated liver enzymes, low platelet count; NICU, neonatal intensive care unit; PPROM, preterm premature rupture of membranes; PROM, premature rupture of membranes.

Bold font (numbers): Statistically significant *P*-value <.05.

^*a*^Comparison group is normal-weight women or infants born to normal-weight women.

^*b*^Data are presented as n (%) with the following exceptions: age (years), birthweight (grams), and gestational age (weeks) are reported as median (25th-75th percentile).

^*c*^Severe preeclampsia/HELLP (n = 551) also includes women with eclampsia (n < 5) and chronic hypertension with preeclampsia (n < 100) due to small sample size of each condition.

^*d*^Fisher's exact *P*-values were used in the case of small sample sizes.

### Obstetrical Complications

In unadjusted models, there was a decreased risk of PROM in low-weight compared with normal-weight women without observed differences in all other adverse pregnancy outcomes (Table 2). The decreased risk of PROM (*P* = .02) in low-weight women persisted after adjustment for confounders (aOR: 0.84; 95% CI 0.73, 0.97) (Table 2). Additionally, low-weight women had a decreased risk of post-partum hemorrhage (*P* = .04) compared with normal-weight women (aOR: 0.73; 95% CI 0.54, 0.98) (Table 2). No other differences were noted.

**Table 2.** Association between adverse pregnancy and neonatal outcomes and underweight maternal body mass index

Adverse outcomes	No. of observations (Unadjusted)	Unadjusted OR (95% CI)	No. of observations (Adjusted)	Adjusted OR*^a^* (95% CI)
Obstetrical complications	16 361		13 060	
Gestational diabetes mellitus	16 361	0.93 (0.76, 1.15)	13 060	0.88 (0.68, 1.11)
Preeclampsia/eclampsia	16 361	0.98 (0.84, 1.14)	13 060	0.95 (0.80, 1.14)
PROM	16 361	**0.88** (**0.78, 1.00)**	13 060	**0.84** (**0.73, 0.97)**
PPROM	16 360	1.02 (0.85, 1.23)	13 059	0.88 (0.69, 1.11)
Postpartum hemorrhage	16 361	0.90 (0.72, 1.11)	13 060	**0.73** (**0.55, 0.98)**
Neonatal outcomes	16 361		13 060	
Low birth weight (<2500 grams)	16 144	**1.34** (**1.21, 1.49)**	12 900	**1.16** (**1.02, 1.32)**
Preterm birth (<37 weeks)	16 358	1.10 (0.97, 1.23)	13 058	1.04 (0.91, 1.19)
SGA*^b^*	15 348	**1.37** (**1.25, 1.50)**	12 259	**1.22** (**1.10, 1.36)**
LGA*^c^*	13 995	**0.54** (**0.40, 0.74)**	11 198	**0.55** (**0.38, 0.80)**
NICU admission	16 151	1.05 (0.94, 1.16)	12 904	0.95 (0.84, 1.07)
Fetal death	16 361	0.97 (0.62, 1.52)	13 060	0.95 (0.57, 1.58)

Abbreviations: CI, confidence interval; LGA, large for gestational age; NICU, neonatal intensive care unit; OR, odds ratio; PPROM, preterm premature rupture of membranes; PROM, premature rupture of membranes; SGA, small for gestational age.

Bold font (numbers): Statistically significant *P*-value <.05.

^*a*^Logistic regression analyses controlled for maternal age, race and ethnicity, marital status, private insurance, smoking during pregnancy, depression during pregnancy, parity, and prepregnancy diabetes and hypertension; comparison group is normal-weight women for pregnancy complications or infants born to normal-weight women for neonatal outcomes.

^*b*^SGA was defined as less than 10th percentile using the Alexander National Fetal Growth Curve [33].

^*c*^LGA was defined as greater than 90th percentile using the Alexander National Fetal Curve [33].

### Neonatal Outcomes

In unadjusted models, infants born to low-weight women had a decreased risk of being LGA but an increased risk of being low birth weight and SGA compared with infants born to normal-weight women (*P* < .01 for all; Table 2). The differences in LGA (*P* < .01), low birth weight (*P* = .02) and SGA (*P* < .01) between infants born to low-weight vs normal-weight women remained statistically significant after adjustment for potential confounders (Table 2).

## Discussion

We evaluated obstetrical complications (ie, PROM, PPROM, preeclampsia/eclampsia, gestational diabetes mellitus, postpartum hemorrhage) and adverse neonatal outcomes (ie, SGA, low birth weight, preterm birth, LGA, NICU stay, fetal death) in a carefully phenotyped population of low- and normal-weight mothers and their respective singletons and found that neonates born to low-weight mothers had an increased risk of SGA and low birth weight and a decreased risk of LGA when compared with those born to normal-weight mothers. Additionally, low maternal BMI was associated with a decreased risk of postpartum hemorrhage and PROM but not PPROM. Our results indicate that infants born to women with underweight BMI during pregnancy are at greater risk of adverse neonatal outcomes, while low-weight mothers have a lower risk of pregnancy complications when compared with their normal-weight counterparts.

Our findings of no or decreased risk of pregnancy complications, including no increased risk of gestational diabetes, preeclampsia, or PPROM, and a decreased risk of postpartum hemorrhage in low-weight compared with normal-weight women have been described previously both in BMI [12, 17, 19, 34-37] and eating disorder cohorts [5, 26, 28, 38-40]. However, the decreased risk of PROM in underweight women is not previously described. Interestingly, PROM but not PPROM was associated with maternal underweight BMI, although a trend for a decreased risk of PPROM was noted after adjustment (Table 2). In our study, approximately 10% of low-weight women compared to 12% of normal-weight women experienced PROM, while rates were comparable for PPROM (4% for both). A possible explanation for the decreased risk of PROM observed in low-weight women in our study is that our low-weight group may be comprised of women with fewer comorbid conditions compared with studies that limited their exposure groups to women with specific eating disorder diagnoses and related hospitalizations, accounting for greater disease severity. However, we cannot exclude the possibility of unmeasured confounding as our models do not account for a history of PROM or PPROM and preterm birth, along with sexually transmitted infections in pregnancy, which are other known risk factors for PROM and PPROM [41] in addition to smoking during pregnancy, sociodemographics, and parity. Also, a decreased risk of postpartum hemorrhage in the low-weight vs normal-weight mothers might be mediated by mode of delivery, as cesarean delivery is a risk factor for postpartum hemorrhage [42]. Preeclampsia and gestational diabetes mellitus are complications more commonly associated with maternal overweight and obesity [12]. Positive associations between preeclampsia and gestational diabetes mellitus and BMI remain nondisputed in populations with increasing BMI, but interestingly, underweight mothers do not have a significantly lower risk of gestational diabetes mellitus [19, 36] and preeclampsia [17, 19, 36], which is consistent with our findings.

Singletons born to underweight women were at increased risk of low birth weight and SGA but decreased risk of LGA, which is consistent with previous systematic reviews and meta-analyses [1, 12, 43]. The independent association observed between maternal underweight BMI and measures of fetal growth restriction, such as low birth weight and SGA, is likely multifactorial but potentially best explained by poor maternal nutritional status [44]. Abnormal nutritional conditions during pregnancy have largely been linked to eating disorders in developed countries, as compared to food deprivation in developing countries [45]. Combined with placental blood supply and fetal growth factors, maternal undernutrition impacts fetal development; deleterious modifications in placental function and fetal epigenetics can occur, thereby leading to such adverse outcomes [46]. More importantly, in epidemiologic studies, metabolic and cardiovascular complications are greater in adults who are SGA at birth compared with adults who are appropriate for gestational age at birth [47]; decreased lean body mass [48], increased insulin resistance [49, 50], and increased carotid intima-media thickness [51] are associated with SGA at birth and likely contribute to the observed associations with type 2 diabetes and hypertension in adulthood [47, 52].

Whether or not a true association exists between maternal underweight BMI and preterm birth remains unclear. In line with Suzuki, Micali et al, and Ekeus et al [26, 28, 29, 34] but not others [1, 4, 5, 12, 38-40, 43, 53-55], no association was observed between maternal underweight BMI and preterm birth. It is possible that we were able to control for potential confounders not available in other studies, such as maternal smoking, race, age, socioeconomic status, and prepregnancy hypertension and diabetes or our study may have lacked sufficient power to detect such differences.

### Strengths and Limitations

To our knowledge, this is the first study to investigate pregnancy/neonatal outcomes in women with an underweight BMI during pregnancy. Prior studies have focused on women with a diagnosis of anorexia nervosa or other eating disorders who may not be underweight at the time of pregnancy or have normal BMIs with inclusion in the low-weight group [14-16, 26-30]. Therefore, this study had several strengths when exploring such outcomes in low-weight mother-infant pairs. First, we sampled from a large, multiracial delivery cohort of women and singleton births, which improved the generalizability of our results; this included using racial-specific BMI cut-offs. Second, we devised clean exposure groups by confirming underweight BMI obtained from the MOMI database with patient records, and therefore, BMI assessments were objective and not self-reported. Third, we were able to control for important sociodemographic and comorbid risk factors, including marital status, health insurance, smoking during pregnancy, and depression, that are not uniformly available in national registry cohorts. Lastly, the use of a normal-weight comparison group rather than mother-infant pairs without a history of anorexia nervosa, as in prior studies, minimized selection bias and overestimation of our results.

Nevertheless, our study is not without limitations. Despite the large sample size, the study is a single-center study, which potentially limits generalizability. Gestational weight gain was also not included as a separate exposure variable, as it would be difficult to accurately assess given the use of BMI during pregnancy as our exposure of interest. Also, although we were able to adjust all our analyses for important potential confounders—such as age, parity, smoking during pregnancy, race, marital status, insurance status, maternal depression, and chronic illness (ie, prepregnancy diagnoses of diabetes mellitus and hypertension)—there are still likely unmeasured potential confounders, including use of assisted reproductive technologies, prior pregnancy loss or miscarriage, history of preterm birth, history of PROM/PPROM, infections during pregnancy, mode of delivery (ie, cesarean vs vaginal delivery), physical activity, and medication use during pregnancy. Additionally, we are unable to comment on fetal growth trajectories in infants born to low-weight vs normal-weight women. Head circumference at birth, which is used to calculate size for gestational age and is impacted by smoking during pregnancy, was not studied.

## Conclusion

Findings from our study of increased risk of low birth weight and SGA—adverse neonatal outcomes—but not maternal-related pregnancy outcomes highlight the importance of an understudied, at-risk group of women with underweight BMI during pregnancy and affected offspring. The increased risk of adverse neonatal outcomes compared with normal-weight counterparts likely emphasizes the importance of targeting a prepregnancy BMI within the normal range, possibly achieved with frequent BMI monitoring and preconception counseling. Also, future steps include delineating causes of maternal underweight BMI, which are not limited to but include constitutional thinness, malabsorptive disorders, and maternal anorexia nervosa, a condition that is often underdiagnosed, as this information can inform future public health strategies to reduce adverse neonatal outcomes in infants born to underweight women. However, the risk of maternal-related complications in underweight women remains uncertain, and our findings underscore the need for prospective studies to assess obstetrical complications in underweight vs normal-weight women matched on risk factors not evaluated by our study, such as cesarean delivery, history of preterm birth, history of PROM/PPROM, and prior pregnancy loss. Also, offspring outcomes beyond the antenatal and delivery period in those born to low-weight mothers, particularly with respect to long-term cardiometabolic complications, need further investigation given the association between SGA and type 2 diabetes and hypertension [56-58].

## Data Availability

Some or all datasets generated and/or analyzed during the current study are not publicly available but are available from the corresponding author on reasonable request.
